# Comparison of Liver Fat Indices for the Diagnosis of Hepatic Steatosis and Insulin Resistance

**DOI:** 10.1371/journal.pone.0094059

**Published:** 2014-04-14

**Authors:** Sabine Kahl, Klaus Straßburger, Bettina Nowotny, Roshan Livingstone, Birgit Klüppelholz, Kathrin Keßel, Jong-Hee Hwang, Guido Giani, Barbara Hoffmann, Giovanni Pacini, Amalia Gastaldelli, Michael Roden

**Affiliations:** 1 Institute for Clinical Diabetology, German Diabetes Center at Heinrich-Heine University, Düsseldorf, Germany; 2 Department of Endocrinology and Diabetology, Heinrich-Heine University Düsseldorf, Düsseldorf, Germany; 3 Institute for Biometrics and Epidemiology, German Diabetes Center at Heinrich-Heine University, Düsseldorf, Germany; 4 IUF – Leibniz Research Institute for Environmental Medicine, Düsseldorf, Germany; 5 National Research Council, Institute of Biomedical Engineering, Metabolic Unit, Padova, Italy; 6 National Research Council, Institute of Clinical Physiology, Pisa, Italy; 7 German Center for Diabetes Research, Partner Düsseldorf, Germany; University of East Anglia, United Kingdom

## Abstract

**Context:**

Hepatic steatosis, defined as increased hepatocellular lipid content (HCL), associates with visceral obesity and glucose intolerance. As exact HCL quantification by ^1^H-magnetic resonance spectroscopy (^1^H-MRS) is not generally available, various clinical indices are increasingly used to predict steatosis.

**Objective:**

The purpose of this study was to test the accuracy of NAFLD liver fat score (NAFLD-LFS), hepatic steatosis index (HSI) and fatty liver index (FLI) against ^1^H-MRS and their relationships with insulin sensitivity and secretion.

**Design, Setting and Participants:**

Ninety-two non-diabetic, predominantly non-obese humans underwent clinical examination, ^1^H-MRS and an oral glucose tolerance test (OGTT) to calculate insulin sensitivity and β-cell function. Accuracy of indices was assessed from the area under the receiver operating characteristic curve (AROC).

**Results:**

Median HCL was 2.49% (0.62;4.23) and correlated with parameters of glycemia across all subjects. NAFLD-LFS, FLI and HSI yielded AROCs of 0.70, 0.72, and 0.79, respectively, and related positively to HCL, insulin resistance, fasting and post-load β-cell function normalized for insulin resistance. Upon adjustment for age, sex and HCL, regression analysis revealed that NAFLD-LFS, FLI and HSI still independently associated with both insulin sensitivity and β-cell function.

**Conclusion:**

The tested indices offer modest efficacy to detect steatosis and cannot substitute for fat quantification by ^1^H-MRS. However, all indices might serve as surrogate parameters for liver fat content and also as rough clinical estimates of abnormal insulin sensitivity and secretion. Further validation in larger collectives such as epidemiological studies is needed.

## Introduction

Hepatic steatosis is the most frequent liver disease in Western countries, closely associates with insulin resistance, visceral obesity, dyslipidemia and type 2 diabetes (T2DM) and is now classified among non-alcoholic fatty liver diseases (NAFLD) in the absence of excessive alcohol intake [1]. The gold standard for diagnosis of NAFLD is the liver biopsy, which is only justified in severe liver disease [2]. ^1^H-magnetic resonance spectroscopy (^1^H-MRS) allows for non-invasive quantification of hepatocellular lipid (HCL) content and for exact diagnosis of steatosis [2], while ultrasound and computed tomography provide rather semi-quantitative estimates [3].

As these techniques are time-consuming, expensive and often unavailable in daily routine, more simple tests have been developed based on routine laboratory and anthropometric parameters. The fatty liver index (FLI) [4], the hepatic steatosis index (HSI) [5] and the NAFLD liver fat score (NAFLD-LFS) [6] yielded satisfying results in their respective collectives, when validated against ultrasound (FLI, HSI) or ^1^H-MRS (NAFLD-LFS). However, despite the association of steatosis with impaired glucose tolerance [7], FLI and HSI seem to perform less well in insulin resistant states such as T2DM [8].

We aimed to test (i) the diagnostic accuracy of these three indices by comparison with exact quantification of HCL by ^1^H-MRS and (ii) the relationships with insulin sensitivity and secretion in a non-diabetic, predominantly non-obese collective of white origin in which median liver fat content is supposed to be low and therefore diagnosis of steatosis appears more challenging. Of note, the FLI has been originally developed to detect steatosis, whereas HSI and NAFLD-LFS have been developed to detect NAFLD. To account for these differences, we also analyzed a subgroup of our collective with low-risk alcohol consumption [9].

## Study Population and Methods

### Study design

This study was performed in the context of the German National Cohort feasibility studies. The protocol is in line with the 1975 Declaration of Helsinki and was approved by the Bavarian Medical Association and the ethical board of Heinrich-Heine University Düsseldorf. All subjects gave their written informed consent to participate.

Overall, from July to October 2011, 148 residents of the Düsseldorf area, aged 22 to 70 years, were recruited from a random sample of the general population. 100 persons agreed to participate in additional clinical examination, blood sampling after 10 hours of fasting, a 2-hours oral glucose tolerance test (OGTT), liver ^1^H-MRS and whole-body MR imaging (MRI). Persons with non-white origin, T2DM and/or with hepatitis B and C were excluded from analysis, because these conditions are known to specifically affect HCL [10] so that 92 subjects remained for further analyses.

### Clinical examination

All participants underwent a structured interview including assessment of mean daily alcohol intake during 7 days using estimated ethanol contents of beverages (beer 5%, wine 12%, shots 40%). The World Health Organization definition was applied for low-risk alcohol (LRA) consumption [9].

Body weight was measured to the nearest 0.1 kg using a calibrated weighting scale (SECA 285; SECA, Hamburg, Germany). Body height and waist circumference (waist) were measured according to standard procedures. Values of systolic (SBP) and diastolic blood pressure (DBP) were measured thrice after 5 min rest in sitting position using a validated automatic device (OMRON HEM 705 IT, OMRON, Mannheim, Germany) and means of the last two measurements were used for analysis.

### Oral glucose tolerance test (OGTT)

A 75 g-OGTT (Accu-Chek Dextro O.G-T., Roche, Basel, Switzerland) was performed after at least 10 hrs overnight fasting. Blood samples were drawn at −5, 30, 60 and 120 min of OGTT and dysglycemia was categorized according to international criteria [11].

### Laboratory measurements

Alanine aminotransferase (ALT), aspartate aminotransferase (AST), γ-glutamyl transpeptidase (γ-GT) and HDL-cholesterol (HDL-C) were measured on a Cobas MODULAR analyzer (Roche, Basel, Switzerland). Triglycerides (TG) were measured on a Hitachi 912 analyzer (Roche, Basel, Switzerland). Blood glucose was measured from venous whole blood samples using an EPOS Analyzer 5060 (Eppendorf, Hamburg, Germany). Insulin was determined by microparticle enzyme immunoassay (MEIA) on an AXSYM analyzer (Abbot, Abbot Park, USA), C-peptide (CP) was measured chemiluminimetrically (Immulite1000, Siemens, Erlangen, Germany).

### ^1^H-MRS and MRI

All measurements were performed using a 3-T MR scanner (Philips achieva, X-series, Eindhoven, Netherlands). For ^1^H-MRS, a stimulated echo acquisition mode (STEAM) sequence (repetition time of 4 s, echo time of 10 ms) was performed on a volume of 3×3×2 cm^3^ in the liver. Spectra was collected without water suppression from 32 acquisitions and analyzed using the NUTS software package (Acorn NMR Inc, Livermore, CA, USA). HCL was quantified and corrected for T2 relaxation times with specific weighting for lipids as previously reported [12], [13]. Steatosis was defined as HCL values ≥5.56% [14]. Whole body MRI was performed to quantify liver volume (HVOL), total (AT_tot_), subcutaneous (SAT) and visceral (VAT) abdominal adipose tissue using transverse multi-slice turbo-spin echo sequences [15].

### Indices of hepatic steatosis

#### NAFLD liver fat score (NAFLD-LFS) [6]



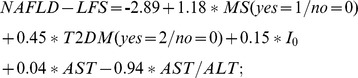
with I_0_ (µU/ml) representing fasting insulin and AST, fasting AST levels (U/l). Values ≤−0.640 rule out, while values >−0.640 rule in NAFLD. Metabolic syndrome (MS) was defined according to the criteria of the International Diabetes Federation [16].

#### Hepatic steatosis index (HSI) [5]



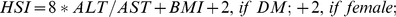
with values <30 ruling out and values >36 ruling in steatosis.

#### Fatty liver index (FLI) [4]



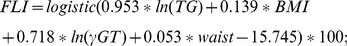
where logistic(x) = 1/(1+e^-x^) denotes the logistic function and ln the natural logarithm. Values <30 rule out and values ≥60 rules in steatosis.

### Index of percentage HCL

#### NAFLD-LFS_cont [6]



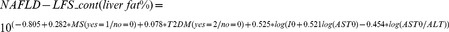
log denotes the decadic logarithm.

### Measures of insulin sensitivity and secretion

#### QUICKI

For fasting conditions, we applied the quantitative insulin sensitivity check index (QUICKI) calculated as 1/[log(G_0_)+log(I_0_)], where G_0_ and I_0_ are fasting glucose and insulin [17].

#### OGIS

Dynamic insulin sensitivity was assessed with the oral glucose insulin sensitivity index (OGIS), derived from a complex mathematical model, which represents total glucose disposal or whole body insulin sensitivity [18].

#### ISIcomp

The Matsuda's index (ISIcomp) was used as another measure of dynamic insulin sensitivity and calculated as  = 10000/√(G_0_×I_0_×G_m_×I_m_), where G_m_ and I_m_ are mean glucose and insulin concentrations during OGTT [19].

#### Fasting β-cell function

During fasting, β-cell function was calculated as CP_0_/G_0_.

#### Insulinogenic indices (IGI)

During glucose loading, the insulinogenic index was calculated as IGI_Ins = (I_30_ – I_0_)/(G_30_ – G_0_), where I_30_ and G_30_ are insulin and glucose concentrations at 30 min of OGTT [20], [21].

IGI_Ins reflects the appearance of insulin in the peripheral circulation.

For more precise assessing of β-cell (pancreatic, pre-hepatic) function, C-peptide levels were used to calculate the IGI_CP as (CP_30_ -CP_0_)/(G_30_ – G_0_), where CP_0_ and CP_30_ are C-peptide concentrations at fasting and 30 min of OGTT [21].

#### Disposition Index (DI)

The DI is given as product of insulin sensitivity (OGIS) with post-hepatic insulin release function (IGI_Ins_tot_) [22], [23].

#### Adaptation Index (AI)

The AI is the product of insulin sensitivity (OGIS) with β-cell function (IGI_CP_tot_) [24], [25].

#### Hepatic insulin extraction

Hepatic insulin extraction was approximated by a function of 1–(AUC_Ins_/AUC_CP_) [26].

### Statistical Analyses

The diagnostic performance of the indices was tested by the area under the receiver operating characteristic curve (AROC) [27]. Confidence bounds for comparison between AROC's were done as described [28]. The Clopper-Pearson method [29] was used to calculate exact confidence bounds for sensitivity (Se) and specificity (Sp) at different cut-off limits. The Youden index was calculated as sum of Se and Sp-1 [30].

Variables with skewed distribution were ln-transformed before correlation and regression analyses. Moreover, the logit transformation (logit(x) = ln(x/(1-x)) was applied to the FLI index, divided by 100, to obtain a corresponding linear (approximately normally distributed) index given by
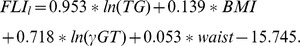



This linear index has identical characteristics (ROC, Se, Sp) as the original index and was only used for regression analysis, for all other analyses we applied the original index.

P-values from two-sided tests less than 5% were considered to indicate statistically significant differences. For comparing two concentration-time curves, we tested specific time points with a Bonferroni-adjusted multiple t-test controlling the family-wise error rate at level 5%. All analyses were performed with SAS for Windows Version 9.2 (SAS Institute, Cary, North Carolina, USA).

## Results

### Clinical characteristics

Persons with steatosis had higher BMI, waist, DBP, TG, ALT, fasting and 2-hour insulin but lower HDL-C *(* *Table 1* *)*. Those with steatosis and low risk alcohol consumption (LRA) also had higher SBP. There were no differences between the respective subgroups with or without LRA.

**Table 1:** Participants' characteristics.

	No steatosis	Steatosis	No steatosis +LRA	Steatosis +LRA
**N (m/f)**	75 (29/46)	17 (7/10)	54 (25/29)	11 (6/5)
**Age (years)**	57.1±12.2	59.9±8.5	56.8±13.1	59.7±9.0
**Alcohol (g/d)**	18.1±16.1	26.1±18.8	11.1±9.7	18.1±12.9
**BMI (kg/m^2^)**	25.3±4.1	28.2±2.8**	25.2±4.1	27.8±2.3§
**Waist (cm)**	87.1±12.4	94.6±8.5*	87.0±12.3	94.7±6.6§§
**SBP (mmHg)**	121.6±15.6	128.1±10.3	122.5±14.3	129.5±6.7§
**DBP (mmHg)**	72.54±8.7	79.3±8.0**	71.9±7.6	80.1±8.9§§
**TG (mg/dl)**	78 [60;117]	109[84;153]*	79[56;110]	125[87;153]§
**HDL-C (mg/dl)**	68.3±17.8	58.1±12.7*	67.3±18.2	55.7±12.7§
**AST (U/L)**	24[21]; [28]	25[22]; [32]	24[21]; [28]	23[21]; [31]
**ALT (U/L)**	18[14]; [25]	26[17;46]**	18[13]; [24]	26[17]; [29] §
**ãGT (U/L)**	20[14]; [30]	30[20]; [35]	21[14]; [30]	30[20]; [35]
**MS (n)**	6	4	3	2
**G_0_ (mg/dl)**	75.7±8.3	77.6±10.7	75.5±7.9	77.2±9.5
**G_120_ (mg/dl)**	89.7±21.5	96.7±26.0	89.7±21.3	92.6±27.7
**I_0_ (µU/ml)**	6[5]; [9]	8[6]; [13] *	6[5]; [9]	8[6]; [12] §
**I_120_ (µU/ml)**	37[24;57]	64[38;119]***	33 [23;57]	67[38;102]§
**Hep_Extr (%)**	69[62;74]	60[57;66]**	69[62;73]	59[56;66]§§
**HCL (%)**	1.3[0.4;3.4]	13.6[8.3;22.3]	1.1[0.4;2.9]	11.8[8.3;20.1]
**HVOL (L)**	1.6[1.4;1.8]	1.8[1.8;1.9]**	1.6[1.4;1.8]	1.8[1.7;1.9]§
**AT_tot_ (L)**	22[18]; [29]	29[27]; [32]***	22[18]; [27]	28[24]; [32] §
**VAT (L)**	2.9[1.7;4.4]	4.3[3.3;6.5]**	3.1[1.6;4.4]	4.6[3.3;6.5]§§§
**SAT (L)**	5.5[4.4;8.1]	7.4[6.6;9.4]	5.4[4.4;7.4]	7.4[5.6;9.4]

Normally distributed data given as mean±standard deviation; Log-normally distributed data as median [25%quartile;75%quartile];

*p<0.05;

**p<0.01 for steatosis vs no steatosis;

§ p<0.05;

§§ p<0.01,

§§§ p<0.001 for steatosis vs no steatosis in LRA.

HCL ranged from 0.03 to 39.01% (median 2.49%; interquartile range (0.62;4.23)) across the whole group (Figure S1 A) and from 0.05 to 30.34% (1.47% (0.60;4.02)) in the LRA subgroup. In the whole group, NAFLD-LFS, HSI and FLI ranged from −4.10 to 2.20, 23.87 to 51.52 and 1.61 to 91.44 with means of −1.81±1.09, 33.71±5.15 and 33.46±26.68, respectively (Figure S1 B,C,D). NAFLD-LFS, HSI and FLI had comparable values in LRA subjects. All indices differed between persons with and without steatosis of the whole group (NAFLD-LFS: p<0.05; HSI: p<0.001; FLI: p<0.01) and LRA subgroups (p<0.01; p<0.01; p<0.05). All indices correlated with HCL and HVOL in the whole and LRA group. HCL and indices also related to AT_tot_, SAT and VAT in the whole and LRA group, except for NAFLD-LFS, which did not correlate with AT_tot_ and SAT (Table 2, for AT_tot_ and SAT data not shown).

**Table 2:** Correlation (R) of HCL and indices with insulin sensitivity, β-cell function, liver volume and visceral adipose tissue.

Variable		HCL_ln	NAFLD-LFS	HSI	FLI
**Liver fat, volume and fat distribution**					
HCL_ln	All	1	0.42***	0.46***	0.50***
	LRA	1	0.26*	0.37**	0.43***
HVOL_ln	All	0.36***	0.38***	0.45***	0.52***
	LRA	0.30*	0.32*	0.39**	0.48***
VAT_ln	All	0.52***	0.52***	0.58***	0.78***
	LRA	0.47***	0.39**	0.54***	0.76***
**Insulin sensitivity**					
ISIcomp_ln	All	−0.46***	−0.71***	−0.53***	−0.62***
	LRA	−0.34**	−0.56***	−0.48***	−0.59***
OGIS	All	−0.46***	−0.51***	−0.50***	−0.62***
	LRA	−0.39**	−0.27*	−0.43***	−0.55***
QUICKI	All	−0.38***	−0.68***	−0.42***	−0.55***
	LRA	−0.24*	−0.62***	−0.35**	−0.46***
**β-cell function**					
DI_ln	All	0.36***	0.57***	0.48***	0.47***
	LRA	0.24	0.46***	0.45***	0.47***
B-cell func_ln	All	0.28**	0.57***	0.47***	0.57***
	LRA	0.10	0.46***	0.45***	0.54***
AI	All	0.22*	0.35***	0.33**	0.34***
	LRA	0.14	0.25*	0.29*	0.29*
IGI_CP_ln	All	0.11	0.05	0.02	−0.02
	LRA	0.08	0.06	0.00	−0.03
IGI_Ins_ln	All	0.22*	0.26*	0.19	0.16
	LRA	0.15	0.25*	0.14	0.11
Hep _Extr_ln	All	−0.34***	−0.55***	−0.42***	−0.39***
	LRA	−0.24	−0.46***	−0.42***	−0.39***

*, p<0.05;

**, p<0.01;

***, p<0.001;

B-cell func, B-cell function.

### Diagnostic performance of indices

**Figure 1:**
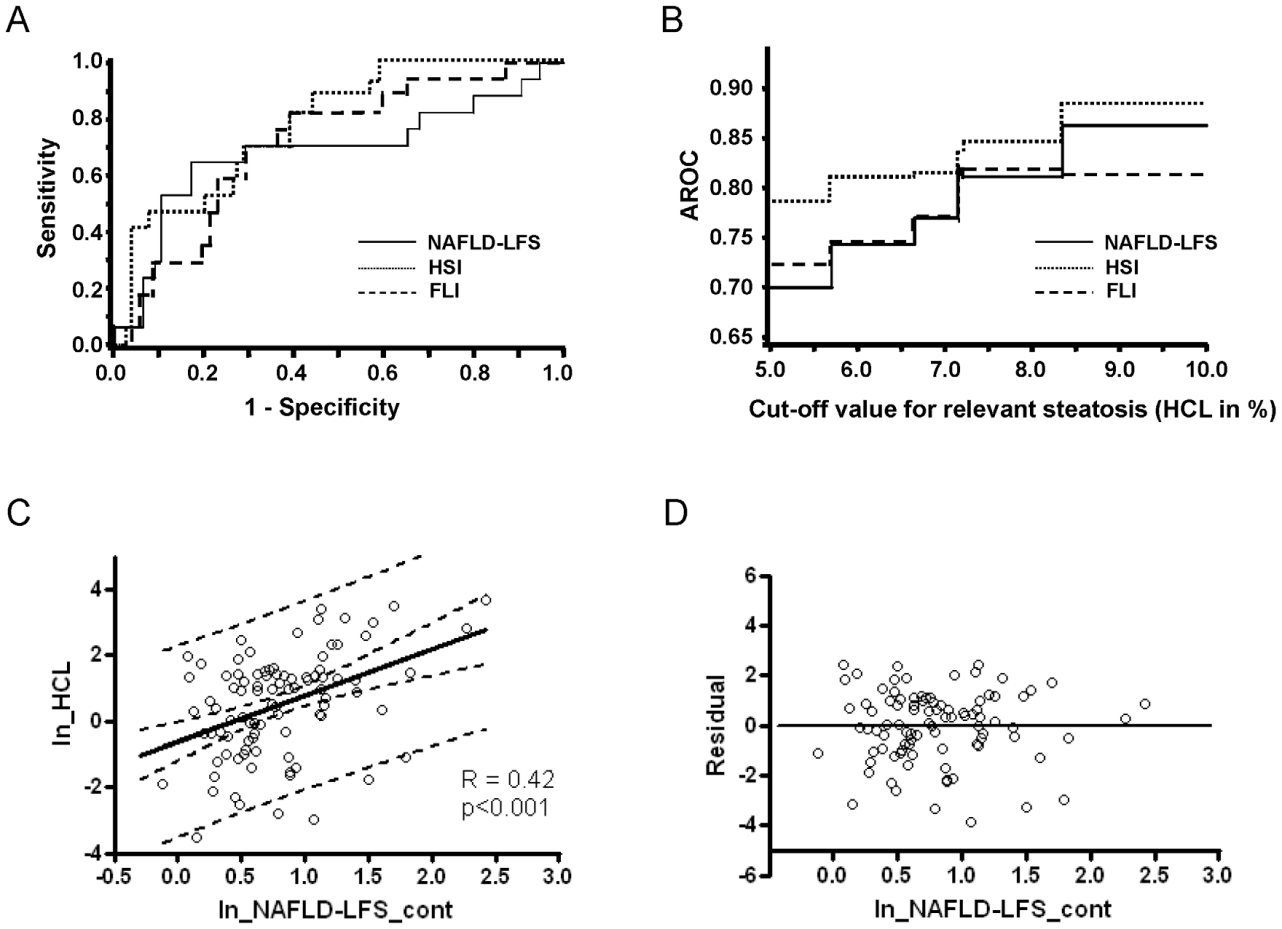
Performance of indices (all subjects). (A) ROC curves of NAFLD-LFS (black line), HSI (dotted line) and FLI (dashed line) (B) AROC's of NAFLD-LFS (black line), HSI (dotted line) and FLI (dashed line) for different HCL cut-offs defining steatosis (C) Correlation of HCL with NAFLD-LFS_cont. Black line, linear regression curve; inner broken lines, 95% confidence limits; outer broken lines, 95% prediction limits (D) Evaluation of goodness of fit by plotting residuals against HCL calculated by NAFLD-LFS_cont.

Across all persons, AROC's were 0.70(95% confidence interval [0.53;0.87]) for NAFLD-LFS, 0.79[0.68;0.90] for HSI, and 0.72[0.59;0.85] for FLI (Figure 1A). In the LRA subgroup, AROC's were 0.75[0.57;0.92], 0.80[0.68;0.92], and 0.75[0.63;0.88], respectively. AROC's did not differ from each other in the whole and LRA group.

Raising the threshold for diagnosing steatosis by HCL above 5.56% improved AROC's for all indices in the whole group (Figure 1B) and in LRA subjects (data not shown). However, AROC of FLI did not further improve at a threshold of 7%.

Applying the originally published cut-off values for each index, which rule in or out steatosis, yielded different diagnostic performance. NAFLD-LFS provided low Se (0.35[0.14;0.62]), but high Sp (0.91[0.82;0.96]). In contrast, HSI had maximal Se (1.00[0.81;1.00]) at the lower cut-off and acceptable Sp (0.75[0.63;0.84]) at the upper cut-off value. FLI had comparable Se (0.76[0.50;0.93]) and Sp (0.83[0.72;0.90]). Analysis of the LRA subgroup revealed similar results (data not shown). We also calculated positive (PPV) and negative predictive values (NPV) of the three indices, with NAFLD-LFS, HSI and FLI having a PPV of 0.46[0.19; 0.75], 0.25[0.16; 0.37] and 0.31[0.18; 0.47]. NPV for NAFLD-LFS, HSI and FLI were 0.86[0.76; 0.93], 0.88[0.77; 0.94] and 0.84[0.73; 0.91], respectively.

To determine optimal cut-off values for each index in our sample, we identified those values that maximize Youden's index. In the whole sample, the optimal cut-off values were −1.02 for NAFLD-LFS yielding a Se of 0.59[0.33; 0.82] and Sp of 0.89[0.80; 0.95], 35.0 for HSI (Se 0.76[0.50; 0.93]; Sp 0.70[0.59; 0.81]), and 29.2 for FLI (Se 0.82[0.56; 0.96]; Sp 0.61[0.49; 0.72]). In the LRA subgroup, the values were −1.12 for NAFLD-LFS (Se 0.64[0.31; 0.89]; Sp 0.87[0.75; 0.95]), 34.0 for HSI (Se 0.91[0.59; 1.0]; Sp 0.67[0.53; 0.79]) and 29.2 for FLI (0.91[0.59; 1.0];0.67[0.53; 0.79]).

After optimization of cut-off values, PPV were 0.56[0.31;0.79] for NAFLD-LFS, 0.37[0.21;0.55] for HSI and 0.33[0.19;0.49] for FLI. NPV were 0.91[0.81;0.96] for NAFLD-LFS, 0.93[0.83;0.98] for HSI and 0.94[0.83;0.99] for FLI. For the LRA subgroup, values were 0.77[0.48;0.95] (PPV) and 0.92[0.81;0.98] (NPV) for NAFLD-LFS, 0.55[0.35;0.74] (PPV) and 0.97[0.86;1.0] (NPV) for HSI, and 0.55[0.35;0.74] (PPV) and 0.97[0.86;1.0] (NPV) for FLI.

Finally, we examined whether specific indices can predict percentage of HCL by applying the previously proposed NAFLD-LFS_cont index using the identical parameters as NAFLD-LFS [6]. NAFLD-LFS_cont correlated with HCL across all (r = 0.42, p<0.001) and LRA persons (r = 0.27, p<0.05) (Figure 1C). However, the differences between observed and predicted ln-transformed HCL values (residuals) ranged from −3.9 to 2.5 (Figure 1D). Translated to the original scale, this means that the ratio of observed and predicted liver fat ranges from 0.02 to 12.2.

### Correlation of HCL and indices with glycemia, insulin sensitivity and β-cell function

**Figure 2:**
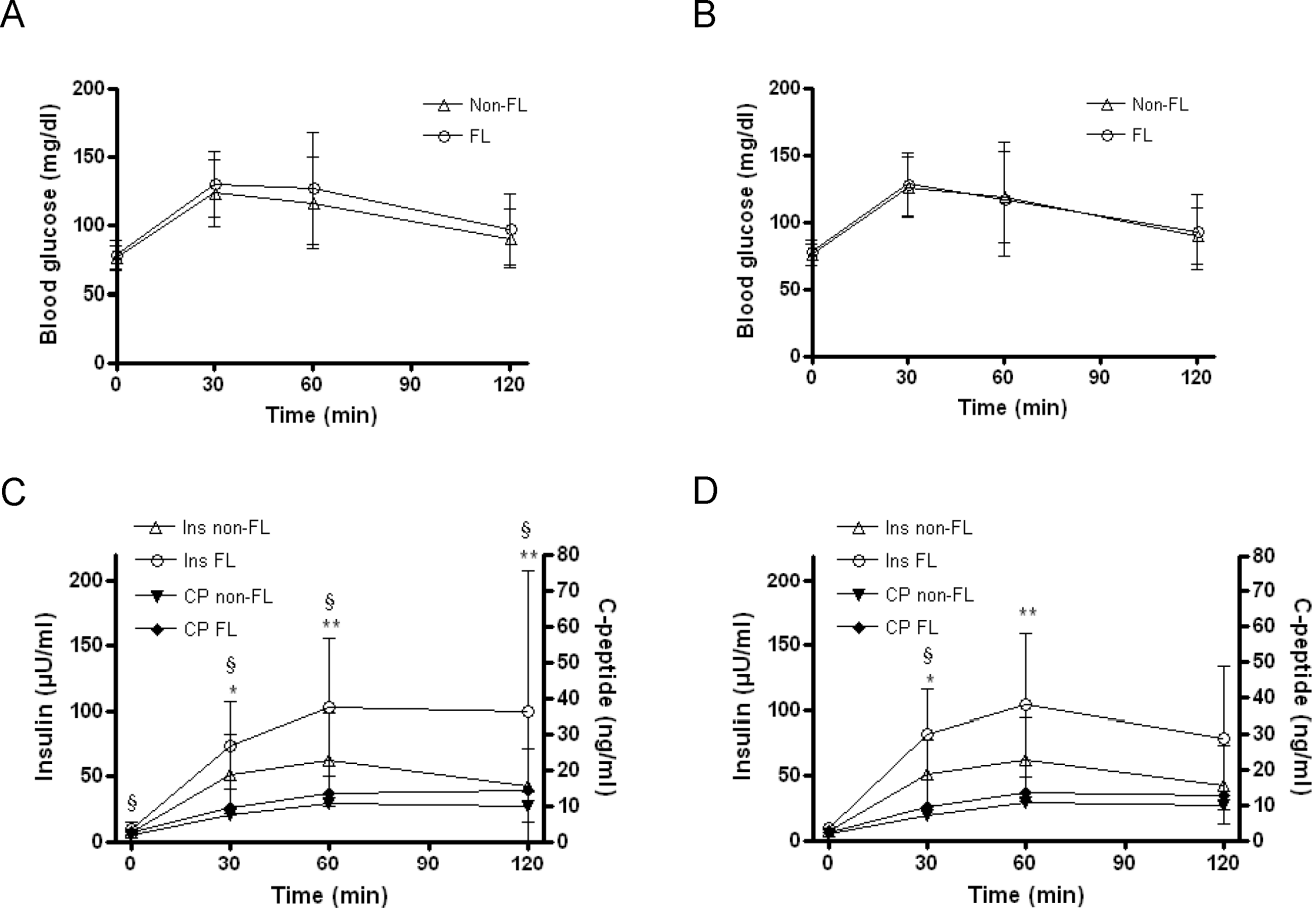
OGTT in subjects with and without steatosis. Plasma glucose (all: A, LRA: B), insulin and C-peptide (all: C, LRA: D) during OGTT in subjects without (non-FL) (insulin: open triangles, C-peptide: black triangles) and with steatosis (FL) (insulin: open circles, C-peptide: black squares). *, p<0.05; **, p<0.01 for insulin; §, p<0.05 for C-peptide.

Subjects with steatosis had similar blood glucose, but higher insulin and C-peptide during OGTT than those without steatosis (Figure 2 A,B). In LRA subjects, presumably due to low sample size, differences in insulin and C-peptide levels were less prominent (Figure 2 C,D).

HCL correlated inversely with fasting (QUICKI) and dynamic insulin sensitivity (OGIS, ISIcomp) and positively with fasting β-cell function and post-load insulin release (DI, AI, IGI_Ins) in all, but not in LRA subjects (table 2).

Also, the indices inversely and strongly correlated with QUICKI, OGIS and ISIcomp (table 2). Even after adjustment for age, sex, and HCL (model-1, table 3) and for LRA (model-2, table 3), FLI, NAFLD-LFS and HSI still related to all parameters of insulin sensitivity.

**Table 3:** Association of indices with insulin sensitivity and β-cell function after adjustment for age, sex, HCL and LRA.

			Model-1 Age,Sex,HCL		Model-2 Age,Sex,HCL,LRA	
		Dependent	Estimate (β)	Partial correlation	Estimate (β)	Partial correlation
**NAFLD-LFS**	All	OGIS	−22.3***	−0.38	−22.9***	−0.39
**HSI**			−4.1**	−0.35	−4.3***	−0.35
**FLI_I_**			−20.4***	−0.46	−21.1***	−0.46
**NAFLD-LFS**		QUICKI	−0.03***	−0.62	−0.03***	−0.64
**HSI**			−0.003**	−0.32	−0.003**	−0.33
**FLI_I_**			−0.02***	−0.45	−0.02***	−0.46
**NAFLD-LFS**		ISIcomp_ln	−0.31***	−0.64	−0.33***	−0.66
**HSI**			−0.04***	−0.41	−0.04***	−0.42
**FLI_I_**			−0.19***	−0.51	−0.20***	−0.52
**NAFLD-LFS**		Disposition Index_ln	0.22***	0.52	0.23***	0.55
**HSI**			0.03***	0.38	0.04***	0.40
**FLI_I_**			0.13***	0.41	0.14***	0.43
**NAFLD-LFS**		Adaptation Index	0.04***	0.34	0.04***	0.36
**HSI**			0.006*	0.24	0.006*	0.24
**FLI_I_**			0.03**	0.32	0.03**	0.33
**NAFLD-LFS**		B-cell func_ln	0.21***	0.53	0.19***	0.53
**HSI**			0.03***	0.40	0.03***	0.40
**FLI_I_**			0.16***	0.56	0.16***	0.56

*, p<0.05;

**, p<0.01;

***, p<0.001;

B-cell func, B-cell function.

In all and LRA subjects, indices correlated with fasting β-cell function, DI and AI. Only NAFLD-LFS related to IGI_INS (p<0.05) (table 2). Applying model-1 on all subjects, correlations between indices and fasting β-cell function, DI and AI were still present. Also, applying model-2, correlations remained (table 3). LRA subjects showed comparable results with model-1, only HSI did not correlate with AI and NAFLD-LFS was not associated with OGIS (data not shown).

Hepatic insulin extraction differed between subjects with and without FL (table 1) and related to HCL and indices in all (p<0.001), but not in LRA subjects (p = 0.06). However, all indices correlated with hepatic insulin extraction in all and LRA subjects (table 2).

## Discussion

In this non-diabetic, predominantly non-obese collective from the general population NAFLD-LFS, HSI and FLI offer a diagnostic efficacy of 70–80% with lower sensitivities and specificities compared with their original description. Interestingly, this study shows additional features of these indices as predictors of insulin resistance and - to less extent - insulin secretion.

Several factors might contribute to the lower than expected diagnostic efficacy of the indices, including selection and characteristics of the study populations (inclusion criteria, risk factor prevalence) as well as measurement technique [27]. In contrast to the populations from which the indices were derived, our study consists of a sample of non-diabetic, predominantly non-obese white persons from the general population. For NAFLD-LFS, the Finnish collective comprises persons without and with T2DM recruited on a 3-to-1 basis for metabolic studies [6]. HSI was derived from data of a Korean cross-sectional case-control study [5]. Finally, FLI however, was developed from data of the Dionysos Nutrition & Liver study, which included residents of Campogalliano in Italy [4], [31], providing a real sampling of general population without particular bias in selection, but development of FLI was based on equally matched persons with and without suspected liver disease (SLD). Comparing these collectives shows marked differences in prevalence of risk factors. The NAFLD-LFS collective presents with already increased risk for metabolic diseases such as T2DM due to greater BMI, BP, TG and transaminases [6]. The HSI collective comprised exclusively Asians, who develop NAFLD at lower BMI with 3.5fold greater prevalence in males, both of which differing from whites [32]. Remarkably, both BMI and sex are variables of the HSI. The FLI collective comprised a white sample of the general population, but cases of SLD were matched with cases without SLD and therefore prevalence of the metabolic syndrome and T2DM might not be the same as in the general population [31].

The sensitivity of ultrasound to detect steatosis is about 91% in patients with HCL ≥30% [33], but only 64% for HCL <30%, indicating that ultrasound misses cases of mild steatosis. Thus, HSI and FLI, which have been validated against ultrasound, may have been rather designed to reliably identify patients with medium to severe fatty liver disease than those with mild steatosis. Testing the accuracy of FLI in a smaller group of women with previous gestational diabetes revealed a strong correlation with HCL measured by ^1^H-MRS [34], whereas FLI and HSI performed less well in patients with T2DM [8]. We found reasonable AROCs for FLI and HSI, but lower diagnostic performance for NAFLD-LFS in our collective. The latter might be due to the lower mean HCL compared to the validation study of NAFLD-LFS [6]. NAFLD-LFS_cont was derived from the NAFLD-LFS collective and developed exclusively to predict percent HCL [6]. The present study showed that the residuals, i.e. the differences between observed and predicted HCL using this specific index, were in most cases as high as the value of HCL contents. This indicates that NAFLD-LFS_cont is not suitable for prediction of HCL at least in non-diabetic collectives with lower prevalence of steatosis. Thus, these scores offer overall modest performance in the clinical setting– even after optimizing cut-off values for our collective. In detail, sensitivity and specificity differ among the three indices between 0.59 and 0.82 for Se and 0.61 and 0.89 for Sp, respectively. This means that up to 41% of the investigated individuals may be classified as patients without FL, although having FL (false-negative rate) and up to 39% of the individuals may be grouped as FL positive, although having no FL (false-positive rate). These data do not support their use as screening tools, at least for populations with similar characteristics as in the present study with such non-obese persons. Additionally, the positive predictive values indicate that in case of a positive test result, the probability that the patient really has FL is only between 33 and 56%. It might be also critical to adjust cut-offs for FL indices for the tested cohort. Nevertheless, the acceptable correlation between fatty liver indices and exact quantification of HCL suggests that these indices might be appropriate surrogate parameters of liver fat content in large epidemiological studies. It is well accepted that hepatic steatosis associates with insulin resistance and hyperinsulinemia even in lean glucose-tolerant subjects [7]. Likewise, FLI correlates with insulin resistance and T2DM incidence [7], [34], [35]. Although NAFLD-LFS also predicted T2DM in a French cohort [36], its relationship with insulin resistance has not been assessed. To our knowledge, HSI has also not been analyzed with regard to insulin sensitivity and secretion. Here we clearly show that all three indices, strongly and inversely correlate with measures of insulin sensitivity.

Of note, less is known on an association between HCL and β-cell function. While HCL and fasting insulin may correlate [37], data on its relationship with dynamic/OGTT postload β-cell function in collectives with normal and impaired glucose tolerance was contradictory [38], [39]. Here, we confirm that HCL relates to various parameters of β-cell function except IGI in all, but not in LRA subjects, and extend this finding to the three indices. The indices only failed to associate with IGI_Ins and IGI_CP, which might result from the pre-described rather low performance of IGI in small- to medium-sized collectives [21].

The novelty of the present study resides in the direct comparison of different indices with HCL measurement by ^1^H-MRS in a single study population of non-diabetic, predominantly non-obese whites and the finding that - while not specific for prediction of hepatic steatosis - they at least partly reflect glucose homeostasis. On the other hand, this study has also certain limitations. First, this study has a rather small sample size and a collective with low mean HCL contents and prevalence of steatosis. This should not influence Se and Sp of the indices [27] and increases the relevance of these results for general screening of steatosis. However, the predominance of persons with low HCL contents might contribute to the wide confidence intervals for sensitivity and the low positive predictive values thereby underestimating the value of the indices. The small sample size may also add to the wide confidence intervals for AROCs of the tested indices. Moreover, when comparing AROCs of the different indices, we did not find significant differences in performances, but we cannot fully exclude that there might be differences in performance we cannot detect with our collective. Thus, further validation of these indices should be performed in larger cohorts.

Second, participants with significant consumption of alcohol were not omitted from the analysis of the whole collective, as the relative contribution of ethanol intake to the pathogenesis of NAFLD is still uncertain [4]. In their regression models, Bedogni et al. even report no association between ethanol intake and steatosis [4]. Recent data suggest that - despite the potential interactions between alcohol drinking and liver injury - moderate alcohol intake may have paradoxical, favorable and gender-dependent effects also in the liver [40], [41]. However, as heavy drinking is known for its deleterious effects on the liver, we set maximum acceptable alcohol intake to 40 g/d for men and 20 g/d for women, which is below the levels set for heavy drinking (>60 g/d for men and >40 g/d for women), for analyses of the LRA subgroup. Of note, all analyses were also performed in this LRA subgroup, which gave similar results as reported for the whole group.

In conclusion, the tested fatty liver indices offer modest efficacy to detect steatosis and cannot substitute for exact fat quantification by ^1^H-MRS. However, they might serve as surrogate parameters for liver fat content and also as rough clinical estimates of abnormal insulin sensitivity and secretion. Further validation in larger collectives such as epidemiological studies is needed.

## Supporting Information

Figure S1 **Comparison of HCL and indices in subjects with and without steatosis.** Box plots of HCL (A), NAFLD-LFS (B), HSI (C) and FLI (D) scores.
(TIFF)
