# Supplementary figures and images for: Comparison of Liver Fat Indices for the Diagnosis of Hepatic Steatosis and Insulin Resistance
Source: PLoS One. 2014 Apr 14;9(4):e94059. doi: 10.1371/journal.pone.0094059 (PMC3986069; doi:10.1371/journal.pone.0094059)

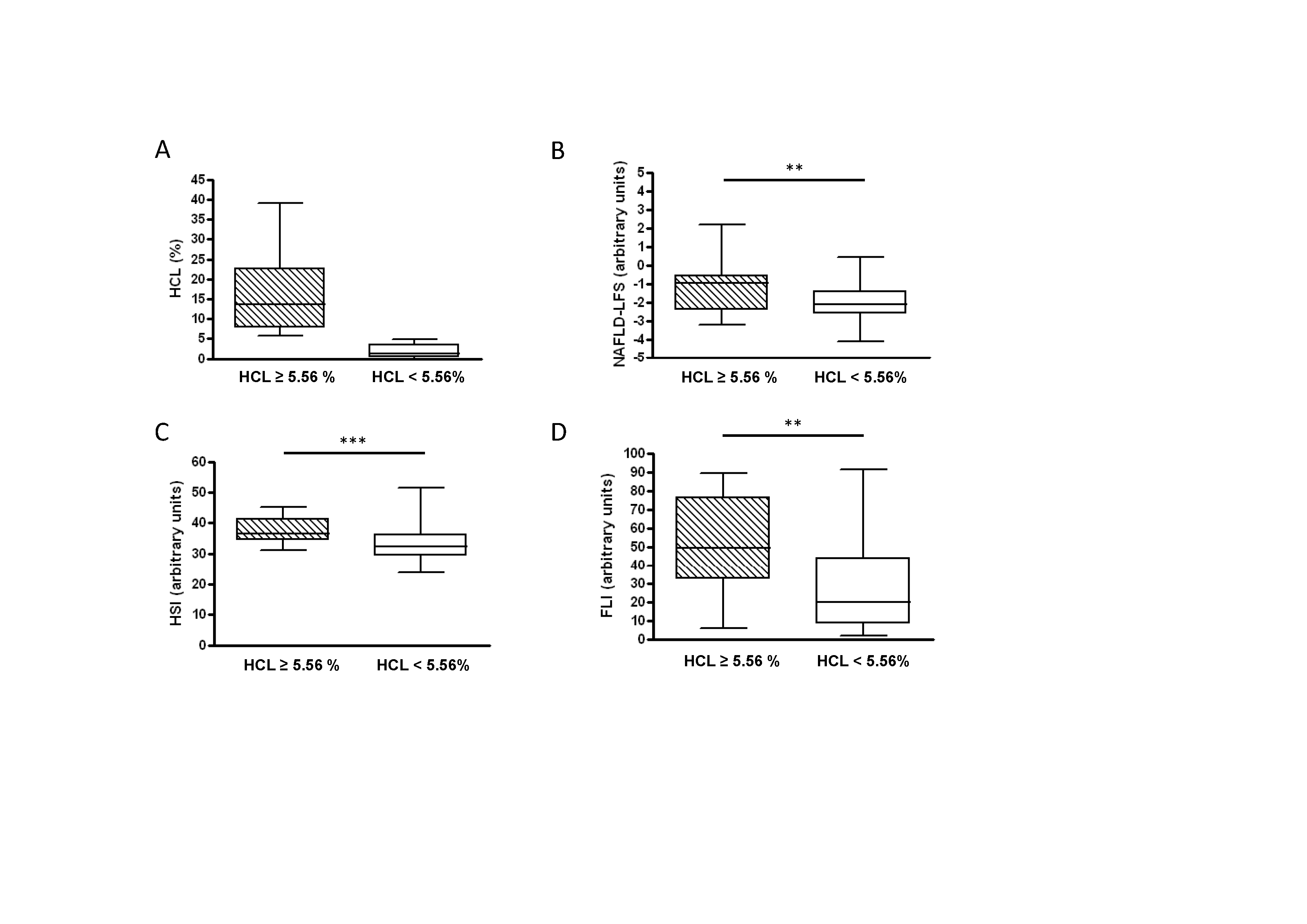

Supplement: Figure S1 — Comparison of HCL and indices in subjects with and without steatosis. Box plots of HCL (A), NAFLD-LFS (B), HSI (C) and FLI (D) scores. (TIFF) [file pone.0094059.s001.tiff]
